# No Mitochondrial Related Transcriptional Changes in Human Skeletal Muscle after Local Heat Application

**DOI:** 10.3390/ijerph192417051

**Published:** 2022-12-19

**Authors:** Monica Kwon, Larry Robins, Mark L. McGlynn, Christopher Collins, Elizabeth J. Pekas, Song-Young Park, Dustin Slivka

**Affiliations:** 1 School of Health and Kinesiology, University of Nebraska at Omaha, Omaha, NE 68182, USA; 2 School of Integrative Physiology and Athletic Training, University of Montana, Missoula, MT 59812, USA

**Keywords:** mitochondrial biogenesis, mitophagy, hot, mRNA, temperature, blood flow

## Abstract

The purpose of the study is to determine the impact of local heating on skeletal muscle transcriptional response related to mitochondrial biogenesis and mitophagy. Twelve healthy subjects (height, 176.0 ± 11.9 cm; weight, 83.6 ± 18.3 kg; and body composition, 19.0 ± 7.7% body fat) rested in a semi-reclined position for 4 h with a heated thermal wrap (HOT) around one thigh and a wrap without temperature regulation (CON) around the other (randomized). Skin temperature, blood flow, intramuscular temperature, and a skeletal muscle biopsy from the *vastus lateralis* were obtained after the 4 h intervention. Skin temperature via infrared thermometer and thermal camera was higher after HOT (37.3 ± 0.7 and 36.7 ± 1.0 °C, respectively) than CON (34.8 ± 0.7, 35.2 ± 0.8 °C, respectively, *p* < 0.001). Intramuscular temperature was higher in HOT (36.3 ± 0.4 °C) than CON (35.2 ± 0.8 °C, *p* < 0.001). Femoral artery blood flow was higher in HOT (304.5 ± 12.5 mL‧min^−1^) than CON (272.3 ± 14.3 mL‧min^−1^, *p* = 0.003). Mean femoral shear rate was higher in HOT (455.8 ± 25.1 s^−1^) than CON (405.2 ± 15.8 s^−1^, *p* = 0.019). However, there were no differences in any of the investigated genes related to mitochondrial biogenesis (*PGC-1α*, *NRF1*, *GAPBA*, *ERRα*, *TFAM*, *VEGF*) or mitophagy (*PINK-1*, *PARK-2*, *BNIP-3*, *BNIP-3L*) in response to heat (*p* > 0.05). These data indicate that heat application alone does not impact the transcriptional response related to mitochondrial homeostasis, suggesting that other factors, in combination with skeletal muscle temperature, are involved with previous observations of altered exercise induced gene expression with heat.

## 1. Introduction

Previous research has shown several associations between mitochondrial dysfunction and the development of diseases such as obesity [1], aging [2], and other neurodegenerative diseases [3]. The development of these diseases highlights the importance of improving mitochondrial health. The growth (biogenesis) and breakdown (mitophagy) of mitochondrial content within skeletal muscle is dependent upon the cellular acute transcriptional response. Acute endurance exercise can initiate the transcriptional potential to increase mitochondrial content in skeletal muscle [4]; however, individuals with mitochondrial-related diseases may have a limited exercise capacity. Indeed, there is a need to explore therapeutic targets to optimize mitochondrial health.

Mitochondrial growth is largely regulated by the *peroxisome proliferator-activated receptor-gamma coactivator 1-alpha* gene (*PGC-1α*), which has been referred to as the master regulator of mitochondrial biogenesis [5]. In addition to *PGC-1α*, a variety of genes for downstream transcription factors are critical to mitochondrial development. These regulatory metabolic genes associated with mitochondrial function include *nuclear respiratory factor 1* (*NRF1*), *GA Binding Protein alpha* (*GABPA*), and *estrogen-related receptor alpha* (*ERRα*). These factors regulate the duplication of mitochondrial DNA through the *mitochondrial transcription of factor A* (*TFAM*) [6] and the activation of the *vascular endothelial growth factor* (*VEGF*) [7,8,9]. Additionally, the accumulation of environmental stressors and oxidative damage may lead to mitochondrial DNA mutations [10] and changes in mitochondrial membrane potential, emphasizing the need for mitophagy by removing damaged mitochondria [11]. The mitophagy associated genes; *PTEN Induced Kinase 1* (*PINK-1*), *Parkin RBR E3 Ubiquitin Protein Ligase* (*PARK-2*), *BCL2 Interacting Protein 3* (*BNIP-3*) and *BCL2 Interacting-Like Protein 3* (*BNIP-3L*) are key in this process. The increased expression of *PGC-1α* mRNA and other genes associated with mitochondrial development are indicators of mitochondrial growth [12] and thus, of interest in the study of mitochondrial development.

Temperature appears to play a role in mitochondrial development of the human skeletal muscle during exercise [13,14,15,16]. However, exposure to environmental temperature extremes without exercise has no impact on the expression of *PGC-1α* [17]. When aerobic exercise is undertaken in the heat, there is a blunted *PGC-1α* mRNA response [13,15,18]. Additionally, the expression of *ERRα* and *NRF1* are also blunted after exercising in the heat when compared to room temperature conditions [15]. Many aspects of mitochondrial related gene expression after exercise in hot environmental conditions has been investigated; however, limited information on the impact of local muscle temperature independent of exercise exist.

Environmental and local temperature alterations are likely to have different mechanisms of action and result in different outcomes for skeletal muscle [13,15,19]. Exercising in the heat increases core body temperature [13,15], whereas applying a local temperature stimulus to a relatively small area has limited impact on whole-body mean skin or core temperatures [13,20]. Thus, by applying local heat on a specific muscle of one limb, while the other serves as control (bi-lateral model), the impact of increased muscle temperature without differentially altering core body temperature can be observed. Local heat application during endurance exercise induces an increase in *TFAM* gene expression; however, other genes related to mitochondrial development may be unaffected [19]. It is currently unknown whether the independent effects of local heat application will induce mitochondrial related transcriptional changes without the presence of an exercise stimulus. As a result, the purpose of this investigation is to determine the effect of acute local heat application (without exercise) on the acute skeletal muscle mitochondrial related transcriptional response related to mitochondrial development.

## 2. Materials and Methods

### 2.1. Study Design

Participants were informed of experimental procedures, risks, and benefits of their participation, followed by a medical history form and a COVID-19 questionnaire. All procedures were approved by the University of Nebraska Medical Center Internal Review Board (IRB #388-20-FB) and conducted in accordance with the declaration of Helsinki. This study recruited 12 recreationally active, healthy individuals between the ages of 19–45 years old (9 males and 3 females; height, 176.0 ± 11.9 cm; weight, 83.6 ± 18.3 kg; and body composition, 19.0 ± 7.7% body fat) that were free from any signs or symptoms of cardiovascular disease or orthopedic limitations.

Participants were instructed to arrive after an overnight fast while avoiding strenuous exercise, alcohol consumption, and tobacco or other drugs for 24 h leading up to the experimental visit. At the beginning of the study, each subject’s height (Seca Standometer; Hamburg, Germany) and weight (Befour Digital Scale, Saukville, WI, USA) were recorded followed by a ten-minute rest period in the supine position. Next, each subject’s body composition was analyzed for descriptive purposes using bioelectrical impedance (InBody Body Composition Analyzer S10, InBody, Cerritos, CA, USA).

Throughout the duration of the experimental visit, the participants rested in a semi-reclined position while thermal wraps, from a fluid circulation-based system, (Cryotherapy ZT Cube, Zamar, Vrsar, Croatia) were wrapped around each thigh for 4 h. In a randomized order, each subject simultaneously received heat application (circulating fluid set to 40 °C) on the experimental limb (HOT) and a wrap with no temperature application (no circulating fluid) on the control limb (CON). After thirty minutes of heat application, each subject consumed a standardized meal of commercially pre-packaged food (638.5 ± 33.1 Kcal, 88.4 ± 8.8 g carbohydrates, 28.2 ± 0.4 g protein, and 21.1 ± 1.1 g fat) and were instructed to consume the meal within 30 min to standardize the dietary state and nutrient intake of each subject. Finally, the 4 h intervention concluded with measures of femoral artery blood flow, skin temperature, intramuscular temperature, and skeletal muscle biopsies from each leg.

### 2.2. Skin Temperature

Immediately after thermal wraps were removed, skin temperature was measured using an infrared thermometer (62 MAX Mini Infrared Thermometer, FLUKE, Everett, WA, USA) and an infrared thermal camera (FLIR C2 Compact Thermal Imager, FLIR Systems, Inc., Wilsonville, OR, USA) on the surface of the HOT and CON *vastus lateralis* (*VL*). The laser thermometer was pointed at the surface of the skin at the approximate site of the upcoming biopsy for ~3 s. The infrared thermal camera captured the emitted infrared radiation of the area of the biopsy sites on both limbs simultaneously. Images were analyzed utilizing the FLIR Tools+ software (FLIR Systems, v.6.0, Wilsonville, OR, USA) by overlaying an ellipse (~7 cm × 15 cm) on both thighs to estimate the average temperature of the *VL*.

### 2.3. Common Femoral Artery Blood Flow and Shear Rate

Doppler ultrasound assessed blood flow velocity and arterial diameter (Terason uSmart 3300, Terason Division Teratech Corporation, Burlington, MA, USA) on the common femoral artery. The common femoral artery was located with the ultrasound probe just distal to the inguinal ligament and proximal to the deep and superficial bifurcations. Vessel diameter was assessed at a perpendicular angle along the central axis of the scanned region. Blood velocities were analyzed in real time (second-by-second) using a frequency of 5 MHz and an insonation angle of 60°. The sample volume was maximized according to the size of the common femoral artery and was centered within the artery. Arterial diameter and mean blood velocity (V_mean_) were recorded. Second-by-second leg blood flow in the common femoral artery was calculated as: blood flow = V_mean_ × π (vessel diameter/2)2 × 60, where blood flow is in mL‧min^−1^ [21]. Shear rate was calculated using arterial diameter and average blood flow velocity as follows: shear rate = (4 × V_mean_/arterial diameter) [22].

### 2.4. Intramuscular Temperature and Biopsies

Each subject had their thighs shaved and cleaned with alcohol. 3 mL of 1% lidocaine was injected into the subcutaneous tissue and fascia adjacent to the *VL* with a 25-gauge needle to anesthetize the area. Betadine, a topical antiseptic bactericide, was spread on the *VL* to disinfect the skin. A sterile field was created with the use of a fenestrated drape, followed by an incision through the skin (~5 mm). Intramuscular temperature was taken through the incision with a hypodermic (~26-guage) thermocouple (MT-26/4HT Physitemp Instruments LLC, Clifton, NJ, USA) inserted ~4 cm into the incision site until readings stabilized. Intramuscular temperature data were recorded using a data logger (Extech Instruments, Nashua, NH, USA). Afterwards, a 14-guage ProMag Ultra Biopsy Needle (ProMag Ultra; Argon Medical Devices Inc., Athens, TX, USA) was inserted into the belly of the muscle at a depth of ~25 mm below the surface of the skin to collect 10–15 mg of muscle. These procedures were then repeated on the other leg. The time between biopsies of the heated and neutral legs was 11 ± 1 min. The delay in biopsy time allowed time to move between limbs. Additionally, the effect of temperature dissipates over time; therefore, the experimental leg was biopsied first to ensure that the muscle sample was collected in the presence of heat. After removal of excess blood, connective tissue and fat, tissue samples were immersed in All-Protect (All-Protect, Qiagen, Hilden, North Rhine-Westphalia, Germany) and stored at 4 °C overnight, followed by storage at −30 °C for later analysis. The muscle biopsies were used to evaluate differences between limbs in specific genes related to mitochondrial biogenesis and mitophagy.

### 2.5. Gene Expression

Real-time reverse transcriptase quantitative polymerase chain reaction (RT-qPCR) was utilized to analyze relative specific mRNA content in the muscle samples. Skeletal muscle tissue (11.2 ± 2.6 mg) was homogenized in 500 µL of Trizol (Invitrogen, Carlsbad, CA, USA) using a handheld homogenizer (Homogenizer 150, Fisher Scientific, Atlanta, GA, USA). Samples were incubated for 5 min at room temperature, 100 µL of chloroform was added, and the tubes were shaken by hand for 15 s. After another incubation at room temperature (2–3 min), the samples were centrifuged at 12,000× *g* for 15 min at 4 °C, and the aqueous layer was transferred to a fresh microcentrifuge tube. Next, 250 µL of isopropyl alcohol was mixed and incubated at −20 °C overnight. The next day, samples were centrifuged immediately at 12,000× *g* for 10 min at 4 °C. The mRNA was washed by removing the supernatant, and adding at least 500 μL of 75% ethanol, and then spun again for 5 min at 10,000× *g*. The resulting mRNA pellet was then dried. Finally, mRNA was dissolved in 30 µL RNA storage solution. The RNA was quantified (222 ± 81 ng·µL^−1^) via Nanodrop (Thermo Fisher Scientific, MA, USA; Wilmington, DE, USA), and RNA quality (RIN: 7.7 ± 0.53) was inspected (2100 Bioanalyzer, Thermo Fisher Scientific, MA, USA; Wilmington, DE, USA), indicating high quality RNA. RNA was converted to cDNA using Superscript IV (Invitrogen) first strand synthesis kit according to manufacturer instructions. Each RT-qPCR 20 μL reaction volume contained 1 μL probe and primer mix (PrimeTime qRT-PCR assay, Integrated DNA Technologies), 10 μL PrimeTime Gene Expression Master Mix (Integrated DNA Technologies), 5 μL deionized water, and 4 μL of sample cDNA. Samples run on an Agilent Technologies Aria Mx real time PCR detection system (Agilent Technologies Inc., Santa Clara, CA, USA) at 1 cycle at 95 °C for 3 min, 40 cycles of 95 °C for 5 s, and 60 °C for 10 s.

The 2^−ΔΔCT^ method was used for the quantification of mRNA for our genes of interest on the experimental limb calculated relative to the control limb and relative to stable reference genes [23]. The most stable reference genes were determined by Normfinder algorithm [24] for each participant. The reference genes used were *Beta Actin* (*ACTB*), Ribosomal Protein S18 (*RPS-18*), *Glyceraldehyde 3-phosphate dehydrogenase* (*GAPDH*), and *Beta-2-Microglobulin* (*B2M*). The geometric mean of the reference genes was used as the stable reference point for each participant. The genes of interest related to mitochondrial biogenesis were total *Peroxisome proliferator activated receptor gamma coactivator 1 alpha* (*PGC-1α*) with defined isoform variance of *PGC-1α-1A*, *PGC-1α-1B*, *NT-PGC-1α* as well as *Nuclear respiratory factor 1* (*NRF-1*), *GA Binding Protein alpha* (*GABPA* or *NFE2L2*), *Mitochondrial Transcription Factor A* (*TFAM*), *Estrogen Related Receptor a* (*ERRα*), Vascular Endothelial Growth Factor (*VEGF*). The genes related to mitophagy were *PTEN Induced Kinase 1* (*PINK-1*), *Parkin RBR E3 Ubiquitin Protein Ligase* (*PARK-2*), *BCL2 Interacting Protein 3* (*BNIP-3*) and *BCL2 Interacting Like Protein 3* (*BNIP-3L*). All probes and primers were designed from Integrated DNA technologies (Prime Time Std qPCR Assay, Integrated DNA Technologies, Coralville, IA, USA).

### 2.6. Statistical Analysis

Excel software (Microsoft v.2211) was used to run paired t-tests to compare means between the control and experimental limbs. 2^−ΔΔCT^ values from mRNA analysis do not follow a normal distribution; thus, statistical analysis of mRNA was performed on log transformed data. A probability of type I error less than 5% was considered significant (*p* < 0.05). All data are reported as means ± SD unless otherwise noted.

## 3. Results

### 3.1. Temperature

**Figure 1:**
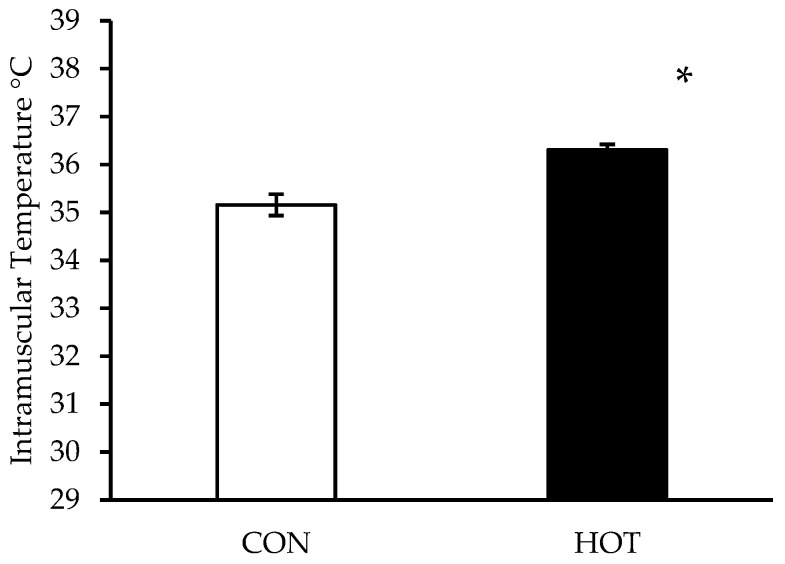
Intramuscular Temperature after 4 h in the control (CON) and heated (HOT) limbs. Data are means ± SD; * *p* < 0.05 from CON.

Skin temperature via laser thermometer was higher in the HOT leg (37.3 ± 0.7 °C) than the CON leg (34.8 ± 0.7 °C, *p* < 0.001). Skin temperature measured with a thermal camera was also higher in the HOT leg (36.7 ± 1.0 °C) than the CON leg (34.6 ± 0.9 °C, *p* < 0.001) throughout the intervention. There was no difference between the single point laser temperature and the elliptical area captured by the thermal camera in the HOT (*p* = 0.397) or CON leg (*p* = 0.102). Intramuscular temperature was higher in the HOT than CON leg (*p* = 0.001) after heat application. See Figure 1.

### 3.2. Common Femoral Artery Blood Flow and Shear Rate

There was no difference in blood velocity at the common femoral artery between the HOT leg (0.67 ± 0.08 m·s^−1^) and CON leg (0.63 ± 0.05 m·s^−1^, *p* = 0.125). There was also no difference in vessel diameter between the HOT (0.62 ± 0.01 cm) and CON (0.62 ± 0.06 cm, *p* = 0.948) legs. Using arterial diameter and mean velocity, the average femoral artery blood flow of the HOT leg was higher (304 ± 35 mL‧min^–1^) than the CON leg (272 ± 40 mL‧min^–1^, *p* = 0.004). Additionally, the average shear rate of the HOT leg (456 ± 71 s^−1^) was higher than the CON leg (405 ± 45 s^−1^, *p* = 0.019).

### 3.3. Gene Expression

**Figure 2:**
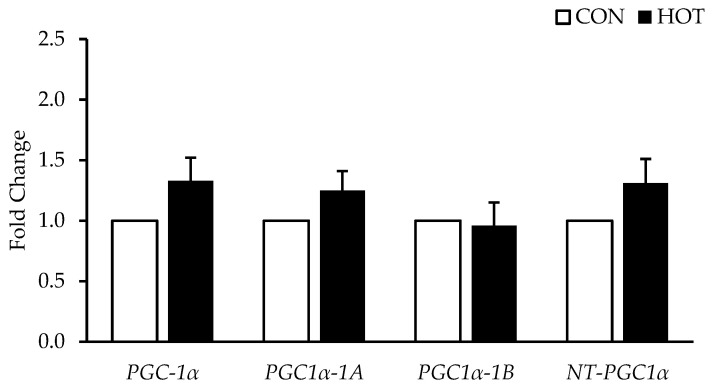
Genes Related to *PGC-1α* mRNA Expression after 4 h in the control (CON) and heated (HOT) limbs. Data are mean ± SEM. HOT is expressed relative to CON (fold change = 1.0).

**Figure 3:**
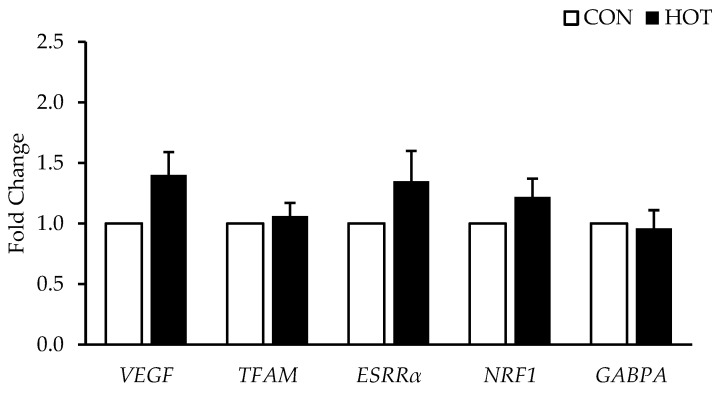
Genes Related to Mitochondrial Biogenesis mRNA Expression after 4 h in the control (CON) and heated (HOT) limbs. Data are mean ± SE. HOT is expressed relative to CON (fold change = 1.0).

**Figure 4:**
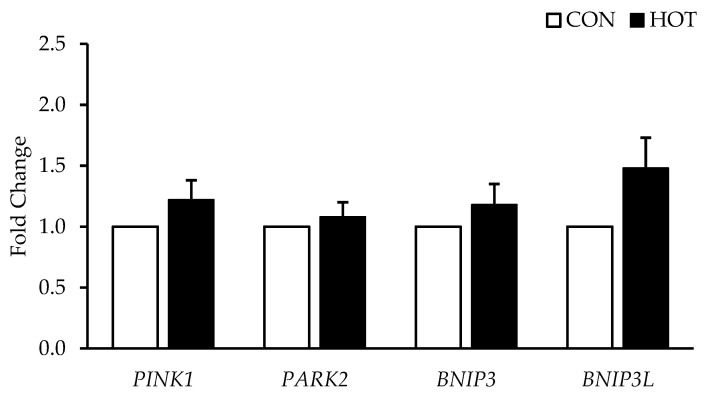
Genes Related to Mitophagy mRNA Expression after 4 h in the control (CON) and heated (HOT) limbs. Data are mean ± SE. HOT is expressed relative to CON (fold change = 1.0).

There were no differences between HOT and CON in any of the *PGC-1α* isoforms (*PGC-1α*, *p* = 0.102; *PGC-1α-1A*, *p* = 0.253; *PGC-1α-1B*, *p* = 0.957; *NT-PGC-1α*, *p* = 0.384). See Figure 2. Additionally, there were no differences in the genes related to mitochondrial biogenesis (*NRF1*, *p* = 0.364; *TFAM*, *p* = 0.914; *VEGF*, *p* = 0.146; *ERRα*, *p* = 0.305; *GABPA*, *p* = 0.297) in response to heat. See Figure 3. Mitophagy related genes were also not different between HOT and CON (*BNIP3*, *p* = 0.469; *BNIP3L*, *p* = 0.147; *PINK1*, *p* = 0.459; *PARK2*, *p* = 0.878). See Figure 4.

## 4. Discussion

The current project investigated the influence of local heat application on skeletal muscle at rest upon gene expression in relation to mitochondrial homeostasis (mitophagy and biogenesis). The results of this study demonstrated that genes related to mitochondrial homeostasis were unaffected by the local heat application. This finding is in agreement with previous work where exposure to ambient heat had no effect on the genes related to mitochondrial biogenesis [17]. However, when heat exposure is paired with exercise, temperature related differences appear [13,14,16,25]. The current project, when taken in conjunction with previous related research [13,17,19], confirmed that exercise is a necessary component to previously observed alterations in mitochondrial related gene expression with temperature interventions. Thus, local muscle heating alone does not elicit significant differences in gene expression related to mitochondrial development. This research provides critical temperature only control data that can aid in the interpretation of previous environmental or local temperature manipulations during exercise.

These molecular methods were limited to the transcriptional response of collected skeletal muscle based on similar gene expression results when combined with exercise. Therefore, without the current work, it is unclear whether observed changes in gene expression were due to temperature exposure alone or its additive/synergistic effects with endurance exercise. Ambient heat and local heat application have differential influences post-exercise upon gene expression; however, regardless of the implementation of heat stimuli, an alteration in gene expression is observed when exercise is present. More specifically, when exercise is coupled with ambient heat exposure, there is a blunted response in *PGC-1α* and other genes associated with mitochondrial biogenesis [13,15,17,18]. In contrast, the application of local heat demonstrated an increased response in *PGC-1α* mRNA after endurance exercise [19]. Past literature, in conjunction with the current study, support the necessity of exercise as the primary stimulus for changes in gene expression. Based on the findings from the current investigation, heat application alone had no effect on the expression of genes related to mitochondrial development and mitophagy. Thus, evidence in human models demonstrate that changes due to heat application are dependent on the presence of an exercise stimulus.

Consistent with previous local heat experimental protocols [13,19], the application of local heat increased skin and intramuscular temperature in the heated limb. The application of local heat to the surface of the skin focuses the transfer of heat energy (i.e., conduction). Energy is transferred by heating up the surrounding particles and penetrating past the subcutaneous fat into the muscle tissue. The heated muscle (36.3 °C) in the current project is similar to what may be expected with light to moderate endurance exercise [19,26]. There are well-known responses of the circulation due to local heat, (i.e., redistribution of blood flow to the skin and muscle), which appears to be temperature-dependent [27]. Heating the quadricep (37 °C) can increase *VL* temperature (+2.8 °C) and femoral artery blood flow (+0.49 L·min^−1^) [27]. In the current investigation, there was an increase in femoral artery blood flow and mean shear rate along with a relatively small, but significant increase (1.2 °C) in intramuscular temperature. When compared to exercise, the blood flow magnitude of change, relative to light-intensity exercise (427.4 mL·min^−1^), [28] is higher than the current 4 h heating protocol (304 mL·min^−1^). These data further confirm that blood flow and mean shear rate increase due to local heat at rest but at a lower magnitude than what would be expected with light exercise. However, populations with limited exercise capacities (i.e., peripheral artery disease) can present with mitochondrial dysfunction [29] and thus increased vascular function due to heat application may have beneficial outcomes [30]. However, further work is warranted to determine any acute alterations in mitochondrial related gene expression in those with limited exercise capacities.

Previously, a related study investigated the impact of local heat application during endurance exercise on mitochondrial related gene expression and demonstrated an increase in *TFAM* but no effect on other genes related to mitochondrial homeostasis [19]. *TFAM* increased with exercise after heat application, but not with exercise alone, suggesting that the response was due to heat application [19]. *TFAM* is essential for mitochondrial DNA (mtDNA) maintenance [31,32,33] and when disrupted, *TFAM* can cause mitochondrial dysfunction [31]. The current study demonstrated no significant effect of local heat on the investigated mitochondrial biogenesis genes; more specifically, local heat alone had no significant impact on *TFAM*. This current study, paired with the previous study, suggest that this *TFAM* alteration was induced by exercise paired with local heat application simultaneously and not independently [19]. Interestingly, intramuscular diathermy (high frequency electrical currents) that increases *VL* temperature (+3.9 °C), increases AMPK phosphorylation, but has no effect on PGC1α protein expression [34]. This study, along with these current findings, confirm that local heat application must be paired with exercise in order to induce acute mitochondrial-related transcriptional actions.

Mitophagy targets dysfunctional mitochondria for degradation by modulating several genes (*PINK1*, *PARK2*, *BNIP3*, *and BNIP3-L*). In the current study, local heat application had no influence over mitophagy related mRNA. Previous investigations using local heat during endurance exercise also reported no alterations in these genes [19]. The exact timing of enhanced expression of mitophagy related genes may have been outside the timeline of the current study and others. Indeed, others have documented that mitophagy is not activated in the early stages post-exercise in a human model [35]. While the current study did not incorporate an exercise challenge, only a thermal challenge, it appears that exercise and other interventions (such as heat) may have a delayed or negligible mitophagy response.

## 5. Conclusions

The primary finding of this study is that genes related to mitochondrial homeostasis are unaffected by local heat application. In conjunction with previous research [17,19] these data suggest that an exercise stimulus is a critical component to previously observed temperature enhanced exercise response. This study helps provide the critical control data needed in order to interpret previous work and to design future studies that combine human temperature interventions and exercise. Further research on the mechanism, which was beyond the scope of this investigation, is needed and may extend to complete genomic and proteomic profiling as well as mitochondrial respiration. None-the-less, this study along with previous studies that have taken a targeted transcriptional approach may provide the framework by which to advance this area of research.

## Data Availability

Data presented in this manuscript may be available upon request from the corresponding author.
